# Impacts of Long-term Fertilization on the Molecular Structure of Humic Acid and Organic Carbon Content in Soil Aggregates in Black Soil

**DOI:** 10.1038/s41598-019-48406-8

**Published:** 2019-08-15

**Authors:** Jiuming Zhang, Fengqin Chi, Dan Wei, Baoku Zhou, Shanshan Cai, Yan Li, Enjun Kuang, Lei Sun, Lu-Jun Li

**Affiliations:** 1 Heilongjiang Academy of Agricultural Sciences Postdoctoral Program, Key Laboratory of Soil Environment and Plant Nutrition of Harbin, Institute of Soil and Fertilizer and Environment Resources, Heilongjiang Academy of Agricultural Sciences, Harbin, 150086 China; 2 Institute of Plant Nutrition and Resources, Beijing Academy of agriculture and Forestry Sciences, Beijing, 100097 China; 3 Northeast Agricultural University, Harbin, 150030 China; 4 National Field Observation and Research Station of Agroecosystem in Hailun, Northeast Institute of Geography and Agroecology, Chinese Academy of Sciences, Harbin, 150081 China

**Keywords:** Carbon cycle, Agroecology, Agroecology

## Abstract

Soil aggregates are the basic units of soil structure, and their composition and carbon (C) stability directly affect soil fertility. As cementing agents, humic substances play an important role in the formation and stability of soil aggregates. Long-term fertilization not only changes the structure of humic acid (HA), but also affects the content and stability of organic C in soil aggregates. In this study, based on a long-term fertilization experiment, the relationship between the molecular structure of HA and the stability of organic C in the aggregates was examined. Compared with the non-fertilization control (CK), both the application of organic manure alone (M) and organic manure combined with inorganic fertilizer application (MNPK) increased organic C content in the bulk soil and in HA. In addition, the application of organic manure (M, MNPK) favored the formation of macroaggregates (>0.25 mm) and showed a higher organic C contents of soil aggregates with different sizes than the CK. Moreover, the content of aliphatic C, the ratios of aliphatic C/aromatic C and alkyl C/O-alkyl C was increased with the application of organic fertilizer. A significant negative correlation was observed between aromatic C and organic C content of the aggregates with sizes of >2 mm, 2–0.25 mm, and 0.25–0.053 mm (*P* < 0.05). The findings indicated that organic fertilization treatments (M and MNPK) increased the aliphatic C content of HA, which favored the increase in the organic C content and stability of the aggregates.

## Introduction

Soil aggregates are the structural units of soils formed by agglomeration and cementation of soil particles. Their quality and quantity not only affect soil fertility, but also maintain soil organic matter (SOM), and are important in carbon (C) sequestration. Humic substances (HS) are the cementing agents forming stable aggregates and are also the main substances involved in C sequestration^1^. The persistence of HS is reliant on being confined within the aggregates^2^. Once the aggregates are destroyed, HS will be exposed and decomposed by microorganisms^3^. Humic substances can maintain the stability of soil aggregates, and the interaction between HS and soil aggregates directly determines soil C sequestration^4^. However, the relations between HS and soil aggregates are poorly understood.

Many studies have suggested that microaggregates, especially those in the macroaggregates, play an important role in the formation and protection of C^5–7^. The organic matter in large aggregates mainly plays a role in physical protection, and is mostly derived from plant residues and sensitive to the management measures, with a short cycle^7^. The macroaggregates contain more C than the microaggregates^8^. The organic matter in small aggregates plays a major role in chemical protection and decomposes slowly, which is beneficial for long-term preservation^9,10^. Tisdall *et al*.^11^ indicated that macroaggregates (>0.25 mm) were mainly formed by the cementation of soil roots and hyphae, whereas microaggregates (<0.25 mm) were mainly formed by polyvalent cationic bridges and polysaccharides. The stability of soil aggregates is affected by soil physical structure and SOM, which in turn, is beneficial to the formation and stability of soil structure^12^. Studies have shown that the aliphatic C in the SOM is an important component of the stable soil C pool and thus plays an important role in C stability^13^. The aliphatic components in SOM are mainly fat, wax, and resin, have a major impact on the decomposition of SOM, and thus affect the release of nutrients and plant growth. Alkanes and fatty acids are a special recalcitrant material in soil C pool^14^. The hydrophobicity of aliphatic compounds is important for the stability of organic matter and aggregates^15^. As an active component of soil HS, humic acid (HA) plays a critical role in soil fertility and nutrient supply capacity. The purpose of this study was to examine the relationship between the HA-C components and the change in the organic C content of the aggregates under different fertilizations.

As SOM is a continuum of progressively decomposing organic compounds^16^, SOM stability as an ecosystem property was predominantly dependent on environment rather than molecular structure alone or physical recalcitrance^17^. To explore the controls of chemical or physical properties on SOM stability, a long-term (32 years) fertilization experiment with the same environment background was selected from the Scientific Observation Station of Heilongjiang Arable Land Conservation and Agricultural Environment, Ministry of Agriculture and Rural Affairs of the People’s Republic of China. The molecular structure of soil HA under different long-term fertilization treatments on black soil was studied by using ^13^C nuclear magnetic resonance spectroscopy (^13^C-NMR). The size composition of soil aggregates was also assessed based on the changes in the composition of soil aggregates and organic C content under different fertilization. The objective of this study was to test the relationship between soil HA-C structure and the stability of organic C in soil aggregates.

## Results

### Effects of fertilization on the contents of SOC and HA

**Figure 1:**
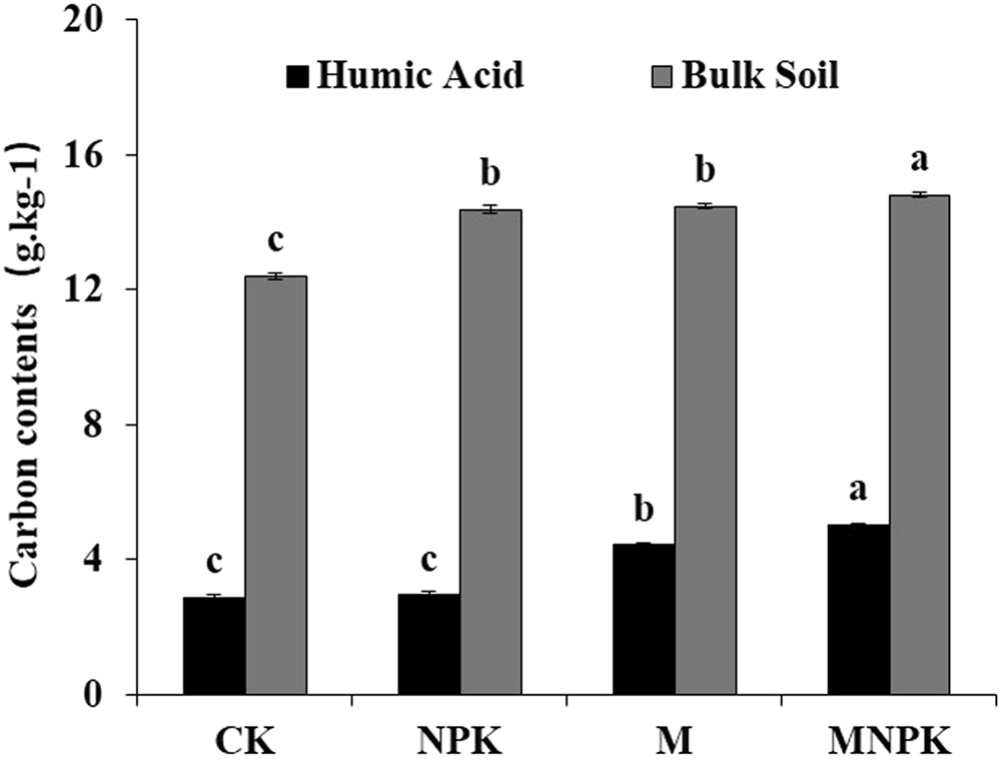
Effects of different fertilization treatments on carbon contents in HA and bulk soil. CK, no fertilizer treatment; NPK, chemical NPK fertilizers; M, organic manure treatment; MNPK, treatment of organic manure combined with chemical NPK fertilizers. Different lowercase letters for each parameter indicate significant difference among different treatments at *P* < 0.05.

Compared with CK, the MNPK, M, and NPK treatments significantly increased SOC (*P* < 0.05) by 19.4%, 16.7% and 15.9%, respectively, and the SOC content of the MNPK treatment was significantly higher than those of M and NPK (Fig. 1). Compared with CK and NPK treatments, the content of soil HA-C was significantly increased by the organic fertilizer treatments (MNPK and M) (*P* < 0.05).

### Effects of fertilization on soil water-stable aggregate composition and organic C content

**Figure 2:**
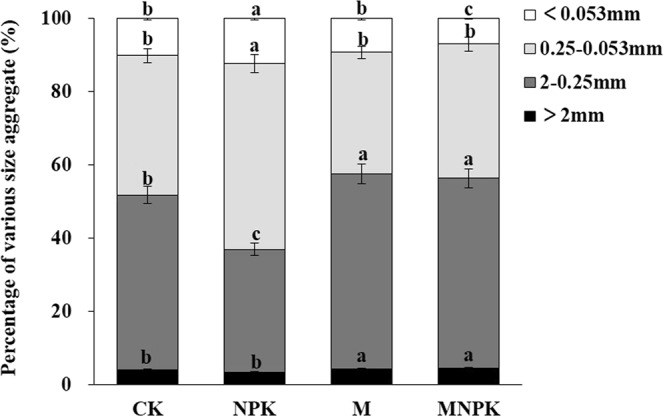
Percentage of the aggregates of all size fractions in the soil under long-term fertilization. CK, no fertilizer treatment; NPK, chemical NPK fertilizers; M, organic manure treatment; MNPK, treatment of organic manure combined with chemical NPK fertilizers.

soil aggregates in the 2–0.25 mm and 0.25–0.053 mm were the dominant soil aggregate size fractions, and the average contents of these fractions across treatments were 46.5% and 39.6%, respectively (Fig. 2). Compared with CK, organic fertilization treatments (M and MNPK) significantly increased the proportions of aggregates in the >2 mm and 2–0.25 mm fractions, and NPK treatment increased the proportions of the aggregates in the 0.25–0.053 mm and <0.053 mm fractions. These results showed that the application of organic manure facilitates the formation of macroaggregates (>0.25 mm).

Generally, the highest organic C content was found in the 0.25–0.053 mm aggregate size fraction, followed by the >2 mm and 2–0.25 mm fractions, and the lowest organic C content was found in the <0.053 mm fraction (*P* < 0.05, Table 1). The increase in organic C content of the aggregates with MNPK treatment ranged from 9.8 (<0.053 mm) to 25.4% (0.25–0.053 mm) relative to the CK treatment. The SOC contents of aggregates under M treatment increased by 14.5–29.0% relative to that of the aggregates in CK treatment, and the largest increase was found in the >2 mm aggregate size fraction. The SOC contents of aggregates with different size fractions under NPK treatment increased by 10.8–18.0% relative to that of the aggregates in CK treatment, and the largest increase was also found in the >2 mm aggregate size fraction.

**Table 1:** Organic carbon contents in soil aggregates of all size fractions (g·kg^−1^ soil).

Treatment	Aggregate size fraction			
	>2 mm	2 mm–0.25 mm	0.25 mm–0.053 mm	<0.053 mm
CK	11.49 ± 0.04 cB	11.61 ± 0.03 cB	13.46 ± 0.04 cA	9.61 ± 0.04 cC
NPK	13.56 ± 0.07 bB	13.49 ± 0.03 bB	15.55 ± 0.05 bA	10.65 ± 0.13 bC
M	14.82 ± 0.08 aB	13.29 ± 0.04 bBC	16.46 ± 0.05 abA	11.32 ± 0.11 aC
MNPK	14.35 ± 0.07 abB	13.76 ± 0.08 aB	16.88 ± 0.05 aA	10.55 ± 0.11 bC

CK, no fertilizer treatment; NPK, chemical NPK fertilizers; M, organic manure treatment; MNPK, treatment of organic manure combined with chemical NPK fertilizers. Different lowercase letters in the same column indicate significant difference among different treatments at *P* < 0.05. Different uppercase letters in the same row indicate significant difference among different aggregate sizes at *P* < 0.05.

### Solid-state ^13^C NMR Spectra and C distribution of HA

**Figure 3:**
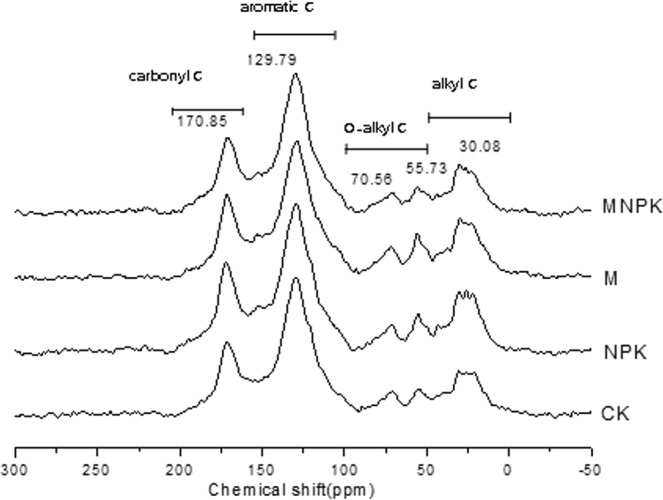
^13^C CPMAS NMR spectra of soil HA. CK, no fertilizer treatment; NPK, chemical NPK fertilizers; M, organic manure treatment; MNPK, treatment of organic manure combined with chemical NPK fertilizers.

The solid ^13^C CPMAS NMR spectra of HA could be divided into four main resonance regions: alkyl C region (0–50 ppm), alkoxyl C (50–110 ppm), aromatic C (110–160 ppm), and carbonyl C (160–200 ppm)^18^. In this study, the main absorption peaks in the resonant regions were near 30, 55, 70, 129, and 170 ppm (Fig. 3). The absorption peaks at 30 ppm is usually assigned to the amorphous (CH_2_)_n_ long chain C, which belongs to the category of alkyl C. The absorptions in the range of 55 to 60 ppm are associated with the Cs in the polypeptide and the methoxy group, and the absorption around 70 ppm is caused by the carbohydrate C^19^. The absorptions in the range of 128 to 132 ppm are mainly designated to the aromatic C with the substitutions of carboxyl group or the carboxyl group and the aromatic group C that is attached to H and is located in the *ortho* position to the O and N substituted groups. The absorptions of 170–172 ppm correspond to the absorptions of carboxylic acid, ester and amide C^20^.

Compared with the CK treatment, the alkyl C contents of HA in the NPK, M, and MNPK treatments increased by 6.8%, 10.5%, and 14.1%, respectively (Table 2). The alkoxyl C contents were decreased in the NPK and MNPK treatments (Table 2). The aliphatic C (alkyl C + alkoxyl C) contents in the M and MNPK treatments were increased by 5.9% and 1.3%, respectively, relative to CK. The aromatic C contents in the NPK, M, and MNPK treatments decreased by 3.2%, 4.3%, and 5.0%, respectively, relative to CK. The carbonyl C contents in the NPK and MNPK treatments increased relative to CK. The application of organic fertilizers without or with chemical fertilizers (M and MNPK) significantly increased the ratios of aliphatic C/aromatic C and alkyl C/O-alkyl C.

**Table 2:** Relative ratios of the functional groups with different chemical shifts from the solid state CPMAS^13^C-NMR spectra of HA in black soil with different fertilization treatments.

Treatment	Alkyl C (%)	O-alkyl C (%)	Aromatic C (%)	Carbonyl C (%)	Aliphatic C/ Aromatic C	Alkyl C /O-alkyl C	Hydrophobic C/ Hydrophilic C
CK	19.1 ± 0.15d	18.4 ± 0.49a	44.3 ± 0.45a	18.3 ± 0.40b	0.84 ± 0.02c	1.04 ± 0.03c	1.73 ± 0.02a
NPK	20.4 ± 0.29c	17.0 ± 0.36b	42.9 ± 0.92b	19.6 ± 0.56a	0.87 ± 0.03bc	1.20 ± 0.03b	1.73 ± 0.07a
M	21.1 ± 0.31b	18.6 ± 0.55a	42.4 ± 0.83b	17.8 ± 0.38b	0.94 ± 0.01a	1.13 ± 0.05b	1.74 ± 0.02a
MNPK	21.8 ± 0.30a	16.2 ± 0.53b	42.1 ± 0.60b	19.9 ± 0.50a	0.90 ± 0.02ab	1.34 ± 0.06a	1.77 ± 0.05a

CK, no fertilizer treatment; NPK, chemical NPK fertilizers; M, organic manure treatment; MNPK, treatment of organic manure combined with chemical NPK fertilizers. Aliphatic C/Aromatic C = (Alkyl C + O-alkyl C)/Aromatic C; Hydrophobic C/Hydrophilic C = (Alkyl C + Aromatic C)/(O-alkyl C + Carbonyl C). Different lowercase letters in the same column indicate significant difference among different treatments at *P* < 0.05.

### Correlation between HA-C structure and the organic C content in soil aggregates

The aromatic C content had a significant negative correlation with the aggregates of >2 mm, 2–0.25 mm, and 0.25–0.053 mm (*P* < 0.05; Table 3). The organic C content in the >2 mm aggregate had a significant positive correlation with that in the 2–0.25 mm (*P* < 0.05) and a significant negative correlation with that of the <0.053 mm aggregate (*P* < 0.05). The organic C content in the 2–0.25 mm aggregate size fraction had a significant negative correlation with that in the 0.25–0.053 mm aggregate size fraction (*P* < 0.05).

**Table 3:** Analysis of the correlation between soil HA-C structural parameters and organic carbon contents in the aggregates.

Factors	Aromatic C	Aliphatic C/Aromatic C	Alkyl C/O-alkyl C	Hydrophobic C/Hydrophilic C	>2 mm	2–0.25 mm	0.25–0.053 mm	<0.053 mm
Aliphatic C	−0.502	0.904	−0.063	0.157	0.676	0.281	0.527	0.768
Aromatic C	1	−0.823	−0.807	−0.691	−0.967*	−0.952*	−0.999*	−0.826
Aliphatic C/Aromatic C		1	0.350	0.423	−0.924	−0.66	0.838	0.924
Alkyl C/O-alkyl C			1	0.838	0.632	0.846	0.796	0.355
Hydrophobic C/Hydrophilic C				1	0.532	0.570	0.699	0.215
>2 mm					1	0.969*	−0.927	−0.959*
2–0.25 mm						1	−0.991*	−0.857
0.25–0.053 mm							1	0.781

Aliphatic C/Aromatic C = (Alkyl C + O-alkyl C)/Aromatic C; Hydrophobic C/Hydrophilic C = (Alkyl C + Aromatic C)/(O-alkyl C + Carbonyl C). * indicates a significant correlation at a 5% level.

## Discussion

### Characteristics of the changes in Soil HA molecular structure

The present study showed that the HA-C structure in the humus of black soil included waxy substance, carbohydrates, carboxyl-substituted aromatic C, carboxylic acids, amide C, some peptides, and methoxy C. The ratio of aliphatic C to aromatic C is usually considered as an index of the humification degree of HA structure^29^. The higher percentages of aliphatic C relative to aromatic C in HA in the treatments M and MNPK (Table 2) indicated that long-term organic fertilizers simplified the structure of HA and decreased the aromaticity. Alkyl C is derived from plant biopolymers (cutin and wax) and microbial metabolites, and is a stable organic C component that is difficult to degrade. In contrast, alkoxyl C is relatively easily decomposed^21^, so the alkyl C/O-alkyl C ratio is usually used as an indicator for the degree of decomposition of organic C^22^. In the current study, the fertilization treatments increased the percentage of alkyl C in HA compared with the CK. The applications of organic manure and inorganic fertilizers allowed more crop residues to enter soils, which likely improved soil microbial activity, and further increased the use of alkoxyl C, causing alkyl C accumulation. In addition, the percentage of alkoxyl C was decreased with the treatment of NPK and MNPK compared to the CK. As a result, the alkyl C/alkoxyl C ratios in all the fertilization treatments were increased relative to the CK, which was beneficial for improving the stability of SOC. The hydrophobic C/hydrophilic C ratio can reflect the degree of hydrophobicity of HS, and is closely related to the stability of the SOC and aggregates. In general, the higher the ratio, the higher the stability of the SOC and the agglomerates. This is because the hydrophobic organic matter containing numerous functional groups, such as –COOH and –OH, can form microaggregates with the charges on the soil mineral surface via hydrogen bonds or polyvalent cation bridges, and the aggregates formed by this cementation have a high stability^22,23^. Giovannini *et al*.^24^ found that hydrophobic organic compounds improved the stability of aggregates and Capriel *et al*.^25^ showed a good correlation between aliphatic organic compounds and the stability of aggregates. The present study showed a slight increasing trend of the hydrophobic C/hydrophilic C ratio in HA with organic fertilization, but there was no statistically significant differences, which deserves further study.

### Relationship between Soil HA-C structure and the Organic C in aggregates

Long-term fertilization has a direct or indirect effect on the formation and stability of soil aggregates, leading to a change in the distribution of soil nutrients in aggregates^26^. As an important component of humus, HA is involved in the distribution of organic C in aggregates, and its structural changes will inevitably affect the distributions of organic C in aggregates. Huang *et al*.^27^ suggested that long chain aliphatic C was a stable C structure in soil, and a higher hydrophobic C/hydrophilic C ratio indicated a higher stability of SOC and aggregates. In the present study, the negative correlation between HA aliphatic C and aromatic C, and that between aromatic C and C content in the >2 mm and 2–0.25 mm and 0.25–0.053 mm aggregates (Table 3) indicated that the decrease of aromatic C content and the increase of aliphatic C content in HA increased the organic C in the agglomerates. Therefore, the increase in aliphatic C content in HA with organic fertilization treatment could help to increase the content and stability of organic C in aggregates. The alkyl C/alkoxyl C and hydrophobic C/hydrophilic C ratios had a positive correlation with organic C contents in aggregates of all size fractions, indicating that all the fertilizations could increase the proportion of organic C that was difficult to decompose in the aggregates and improve the stability of soil C. The above results demonstrated that soil HA structure was correlated to the content of organic C in the aggregates. The organic fertilization treatment could significantly increase SOC and HA contents and increase the proportion of macroaggregates (>0.25 mm). Moreover, the content and stability of organic C in microaggregates (<0.25 mm) were increased because of the increase in the aliphatic C content. Since HA is an important component of humus and is involved in the distribution of organic C in the aggregates, the hydrophobicity of the aliphatic C increases the stability of C and allows microaggregates to contain more organic C in the form of chemical protection, thus improving soil structure and increasing soil C pool.

## Conclusion

Results indicated that soil HA structure was correlated with organic C content in aggregates. M and MNPK treatments significantly increased SOC and HA content, increased the proportion of macroaggregates (>0.25 mm) and aliphatic C content, and then increased the content and stability of organic C in microaggregates (<0.25 mm). As an important component of humus, soil HA participated in the distribution of organic C in aggregates. The hydrophobicity of aliphatic C in HA improved the stability of C, formed chemical protection for soil microaggregates, and increased the content of organic C in microaggregates, thus improving soil structure and increasing soil C pool.

## Methods

### Study site and experiment design

The black soil fertility long-term monitoring station (Scientific Observation Station of Heilongjiang Arable Land Conservation and Agricultural Environment, Ministry of Agriculture), established in 1979 is located in Guangming village of Minzhu town at Harbin, Heilongjiang Province (E126°51′05″, N45°50′30″, 151 m a.s.l.). The station is on the Songhua River secondary stream terrace. The region has a temperate continental monsoon climate and a ≥ 10 °C annual average effective accumulated temperature of 2700 °C, annual precipitation of 533 mm, and a 135-day frost-free season. The soil was developed from loess-like parent material, with the following soil properties in 1979: 15.5 g kg^−1^organic C, 1.47 g·kg^−1^ total nitrogen (N), 1.07 g·kg^−1^ total phosphorus (P) as P_2_O_5_, 25.16 g·kg^−1^ total potassium (K) as K_2_O, 151 mg·kg^−1^ alkaline hydrolyzable N, 51 mg·kg^−1^ available P, 200 mg·kg^−1^ available K, and pH of 7.2.

The long-term experiment was conducted using a wheat (year of 1980)-soybean-maize rotation. Four treatments were arranged with completely random design with three replicates. The size of each plot area was 36 m^2^. The treatments include control (no fertilization, CK), organic manure (M), inorganic N, P, and K fertilizers (NPK), and combined organic manure, inorganic N, P, and K fertilizers (MNPK). The amounts of applied fertilizers in different crop years are shown in Table 4. The organic manure was pure horse manure, collected from the same horse farmers, and applied after the maize was harvested in each rotation at a pure N amount of 75 kg·ha^−1^ (about 18,600 kg·ha^−1^ of horse manure). The contents of organic C, N, phosphorus as P_2_O_5_, and potassium as K_2_O in manure were 163.6 g·kg^−1^, 5.8 g·kg^−1^, 6.5 g·kg^−1^, and 9.0 g·kg^−1^, respectively. The N, P, and K fertilizers were applied in the fall (during maize season, 50% N fertilizer was applied in the fall, and a top dressing of the remaining 50% was performed in the big trumpet period). The N, P, and K fertilizers were urea (N, 46%), tripe superphosphate (P_2_O_5_, 46%), and potassium sulfate (K_2_O, 50%). The detailed information was described by Zhang *et al*.^28^.

**Table 4:** Long-term fertilization experiment and the amount of applied fertilizers.

Treatment	N (kg·ha^−1^ y^−1^)			P_2_O_5_ (kg·ha^−1^ y^−1^)			K_2_O (kg·ha^−1^ y^−1^)	Horse manure (t·ha^−1^)
	Wheat	Soybean	Maize	Wheat	Soybean	Maize		
CK	0	0	0	0	0	0	0	0
NPK	150	75	150	75	150	75	75	0
M	0	0	0	0	0	0	0	18.6
MNPK	150	75	150	75	150	75	75	18.6

CK, no fertilizer treatment; NPK, chemical NPK fertilizers; M, organic manure treatment; MNPK, treatment of organic manure combined with chemical NPK fertilizers.

The tested soil samples were collected at a depth of 0–20 cm after the fall harvest in 2012 (maize) and S-type sampling was adopted, with a total of five sampling sites. After root fragments and stones were removed, a portion of the samples were air-dried and passed a sieve (<2 mm) for further analyses of HA and nutrients.

### HA separation and purification

The detailed method of HA separation and purification was described by our previous study^29^. Briefly, 100 g air-dried soils and 1 mol L^−1^ HCl solution were added into a glass bottle at a ratio soil to solution of 1:1. The ratio was adjusted to 1:10 with HCl solution after standing for 1 h. The mixture was then shaken for 1 h and then centrifuged at a speed of 3500 r min^−1^ for 15 min. After that, the supernatant was discarded and the sediment was adjusted to pH of 7 with 1 mol L^−1^ NaOH solution. After standing for 1 h, the soil to solution ratio was adjusted to 1:10 by adding 0.1 mol L^−1^ NaOH solution. Each glass bottle was flushed with N_2_ for 1 min and then sealed. Under the N_2_ atmosphere, each bottle was shaken once an hour for 12 h, and then settled overnight. Samples were centrifuged at a low speed, acidified to pH 1.0 with 6 mol L^−1^ HCl solution, and then settled again for 12–16 h. Following three extractions and low-speed centrifugations, the sediment was HA. The HA precipitant was dissolved in a small amount of 0.1 mol L^−1^ KOH solution under an N_2_ atmosphere, and then solid KCl was added to achieve a concentration of K+ at 0.3 mol L^−1^. After 1 h settling and centrifugation at a high speed of 8000 r min^−1^ for 20 min, the supernatant was adjusted to pH 1.0 with 6 mol L^−1^ HCl solution, and then settled for 12–16 h. The upper clear solution was discarded. The HA was soaked in 0.1 mol L^−1^ HCl and 0.3 mol L^−1^ HF mixed acid to reduce the content of ash to less than 1%. The samples were then placed into an electrodialyzer until no Cl^−1^ was detected in distilled water by AgNO_3_. The liquid was concentrated by rotary evaporation and then freeze-dried for later use.

### Nuclear magnetic resonance spectroscopy (CPMAS^13^C-NMR) analysis

Humic substances are mainly divided into HA, fulvic acid, and humin. As an active component of soil HS, HA was selected to run the NMR analysis in the present study. The solid-state ^13^C-NMR spectra were collected by nuclear magnetic resonance spectrometer (BrukerAV400, Swiss). The cross-polarized magic angle spin (CPMAS) technique was adopted, the ^13^C resonant frequency was 400.18 MHz, and the magic angle was 8 kHz. The contact time was 2 ms, the recycle delay was 3 s, and the number of data points was 3000. The chemical shift was calibrated by external standard 2, 2**-**dimethyl**-**2**-**silapentane**-**5**-**sulfonate sodium salt, the integral area was automatically given by the instrument, and the relative content of each type of C was expressed by the percentage of an integral area of one chemical shift range over the total integral area.

### Determination of aggregate size and organic C content

Aggregates were graded using the wet sieving method. The air-dried soil sample (100 g) was placed on the top layer of the three-stack (2 mm, 0.25 mm, and 0.053 mm) mechanical sieve shaker. After immersion in distilled water for 5 min at room temperature, the sample was shaken at a frequency of 0.5 Hz and an up-down motion amplitude of 3 cm for 2 min. After sieving, the aggregates on each sieve stack were rinsed into the beaker to obtain water-stable aggregates with particle size >2 mm, 2–0.25 mm and 0.25–0.053 mm, and the aggregate <0.053 mm was settled in the barrel for 48 h and then transferred to the beaker after the supernatant was discarded. The aggregates in the beaker were dried and weighed and the percentage of aggregates in each size fraction was calculated^9^. The SOC content was measured using a total organic C analyzer (Multi N/C 2100S, Analytik Jena, Germany).

### Statistical analyses

The CPMAS^13^C-NMR spectra were analyzed by MestRe Nova (version 6.0.3, Mestrelabs Research, SL, Santiago de Compostela, Spain). After the source data were extracted and analyzed, Microsoft Office Excel 2010 and Origin 8.0 software were used for data processing and plotting, the data in “Available Data” were plotted by fitted curve stacking method in Origin, and SPSS 17.0 statistical analysis software was used to determine whether there were significant differences between groups (Duncan method) and to conduct correlation analyses.
